# Value‐Based Healthcare—What Does it Look Like for Medical Radiation Sciences in the Australian Context?

**DOI:** 10.1002/jmrs.877

**Published:** 2025-03-13

**Authors:** Andrew Davison

**Affiliations:** ^1^ NSW Health St Leonards New South Wales Australia

## Abstract

This editorial provides insight into value‐based healthcare in Australia, including organisations such as New South Wales (NSW) Health, which has a focus on implementing and scaling value‐based healthcare. This includes exemplary programmes in applying value‐based principles in commissioning medical imaging services and expanding hypofractionation radiation therapy for breast cancer patients across the system. This special issue also showcases research in value‐based healthcare and its application in medical radiation sciences in the areas of artificial intelligence (AI), virtual reality (VR), computed tomography (CT) and hypofractionated radiation therapy.

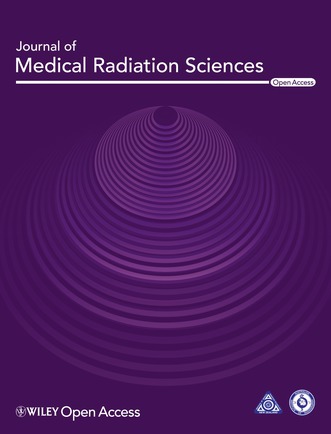

To understand value‐based healthcare, it is important to understand where the concept came from. It was developed by Michael E. Porter, a renowned Professor in business and economics at the Harvard Business School. Porter's academic career has focused on business competitive advantage, and he is famous for creating Porter's Five Forces model for analysing the competitive environment within which a business operates [1]. It is through this lens that Porter and his coauthor, Elizabeth Teisberg, developed value‐based healthcare, which they described in their 2006 book, Redefining Health Care: Creating Value‐Based Competition on Results [2].

Porter and Teisberg observed that healthcare spending in the United States was the highest per capita in the world (and continues to be [3]) and they were achieving poorer health outcomes. They put this down to zero‐sum competition [4] and called for a refocus of healthcare systems on delivering value to patients which, put simply, is improving health outcomes which matter to them. They observed that while many healthcare organisations undertake quality improvement activities, these do not necessarily lead to value. Teisberg argues that value is only achieved when a person's health improves and describes value in health as ‘the measured improvement in a person's health outcomes for the cost of achieving that improvement’ (p 682) [5].

While many of Porter and Teisberg's observations were most relevant to the American healthcare system, focusing on creating value for patients through improving their health outcomes should remain the primary goal for all healthcare organisations across the world. In this context, many of the elements which they described for creating a value‐based healthcare system are transferrable to the Australian setting. These include providing multidisciplinary team‐based care, measuring patient health outcomes and experience of receiving care, integration across primary and secondary healthcare settings, driving more efficient models which deliver the same health benefits and funding mechanisms which focus less on activity and more on outcomes [5].

One approach which has been adopted by many healthcare organisations to support the delivery of value‐based healthcare is the US Institute for Healthcare Improvement's Triple Aim framework [6]. The ‘triple aims’ for healthcare organisations are (1) to improve the health of the population through better patient outcomes and (2) to improve patient experience of receiving care while (3) providing healthcare in a cost‐effective manner. Contemporary variants of this framework also include the importance of clinician well‐being and experience of providing healthcare and addressing health inequities, or the Quadruple and Quintuple Aims, respectively [7].

By focusing on these aims, healthcare organisations are often required to balance achieving patient health outcomes with the cost of delivering care, while ensuring a positive patient experience. Moreover, while patient satisfaction and delivering cost‐effective healthcare are important, Teisberg argues that value is only achieved when patient outcomes are improved and cautions against the overuse of patient experience and reducing costs as signs of value [5]. Despite this, the Triple Aim and its variants are useful models to guide and support healthcare organisations in measuring and achieving their value‐based healthcare goals, and they are also useful in guiding individual clinicians' practice [6].

A focus on value‐based healthcare also draws clinician's and organisation's attention to reducing low‐value care which is essential for delivering a cost‐effective healthcare system, while improving patient health outcomes [8]. Low‐value care is defined as ‘care that confers no benefit or benefit that is disproportionately low compared with its cost, is of low value and potentially wastes limited resources’ (p 179) [9]. Unnecessary treatments, procedures and diagnostics which do not make a difference to the patient's health outcomes or cause harm are arguably the antithesis of value‐based healthcare as they incur health costs for no value. As an example of this, Kamarova et al. [10], who features in this special issue of the Journal of Medical Radiation Sciences, highlights the potential overuse of computed tomography (CT), which may possibly be causing harm at a population level in Australia due to increased exposure to radiation. Kamarova suggests that this may be a result of policy and system factors such as restrictions on the use of magnetic resonance imaging (MRI) and 4‐hr treatment time targets in emergency departments that require rapid diagnostics.

In the Australian context, value‐based healthcare strategies and initiatives have been introduced into state and territory health systems and primary healthcare. In New South Wales (NSW) Health, this has been supported and enhanced through value‐based approaches to commissioning services, improved data sharing across secondary and primary care [11] and the development of tools and guides for measuring value and standardising patient‐reported outcome and experience measures [12]. An example of this is ‘Commissioning for Better Value’ which has been used in Northern NSW Local Health District for commissioning medical imaging services [13]. Through commissioning for better value, rather than adopting a traditional contracting model, the district codesigned the service specifications and contract with clinicians, to focus on value and not just the volume and timeliness of images and reporting. This has resulted in improved patient and clinician experience as well as improved image quality and additional services, while better managing costs.

Other enablers to drive value‐based healthcare has been the implemention of exemplar programmes through the Leading Better Value Care initiatives [14]. One example of this is a programme developed by the Cancer Institute NSW, which aims to increase access to hypofractionation radiotherapy and reduce variation in the treatment of breast cancer [15]. This programme has significant potential to increase value in the healthcare provided by improving the health outcomes of women undergoing radiation therapy for breast cancer, compared with usual care, while also improving patient experience and delivering cost‐effective treatment. Through this programme, the Cancer Institute NSW is supporting Local Health Districts' radiation oncology services with clinical redesign, understanding the enablers and barriers of some patients towards hypofractionation and improving reporting and data sharing across the system [15].

This special issue features two articles which study the growth and benefits of hypofractionation in rural settings in New South Wales [16] and Queensland [17]. Both these studies focused on nonclinical outcomes and showed significant growth in hypofractionation in cancer treatment, particularly for breast and prostate cancers over the past 10–15 years. The benefits of hypofractionation reported by the authors, however, differ. Tabet et al. [17], in their retrospective clinical audit, noted that while there had been a significant uptake in hypofractionation, there had also been an increase in the complexity of treatments, and as such, increasing the need for additional resources to deliver value‐based radiotherapy. Conversely, the literature review undertaken by Yeo et al. [16] identified improvements in productivity and cost savings.

The Yeo et al. [16] review adopted the ‘Triple Bottom Line’ to assess value in the healthcare context. This emerging novel approach to understanding value in healthcare focuses on the social, environmental and economic outcomes of a service or treatment [18]. In doing so, Yeo et al. [16] highlight the benefits, and therefore the ‘value’ of hypofractionation through saving time in patient travel, medical consultations, Linac operating and radiation therapist time. This resulted in reduced healthcare costs and fewer carbon emissions. While these are positive developments for efficiency and time savings, to echo Teisberg's caution [5], it is important to not lose the focus of improving patient health outcomes to truly achieve value‐based healthcare.

The adoption of new technologies offers significant opportunity to progress value‐based healthcare [19]. New technologies, however, come at a cost which may result in a decrease in value compared to the benefits. When introducing new technologies, it is important that these are assessed against value‐based principles, such as the Triple or Quadruple Aim, including clinician experience. This special issue provides two examples where virtual reality (VR) is used in training radiography students in X‐ray examinations [20] and clinicians in radiation safety [21]. While one could assume that VR would have benefits in both these scenarios, fewer benefits were observed in training students, while the use of VR in training clinicians resulted in very positive outcomes.

Likewise, there is significant potential in using artificial intelligence (AI) to improve value‐based healthcare [22]. AI is being introduced in many areas of medical radiation science, with the potential to reduce costs and improve accuracy and patient outcomes. The study featured in this issue identified some of these benefits, particularly in time savings, as preferred by radiation therapists. It also highlighted, however, the risks of algorithmic bias that may result in reducing value‐based health due to poorer patient outcomes and the importance of AI systems that provide explanations for the decisions made [23].

Finally, value‐based healthcare should not just be the domain of healthcare organisations but is important for all health practitioners to embed in their clinical practice. By applying the Quadruple and Quintuple Aims [7] into their practice as a mechanism for delivering value‐based healthcare, a practitioner's primary focus should be to ensure (and measure) that the care and treatment they deliver to individual patients is improving the health outcomes which matter to them. They should identify individual patients and cohorts where there is disadvantage, vulnerability and inequity and adjust their practice (and costs) to address their needs [7]. Practitioners should also continue to check in with patients about their satisfaction and experience of receiving care, and also ensure that as health practitioners they continue to enjoy their clinical practice and experience of delivering care. In this way, Teisberg highlights that value‐based healthcare has a significant benefit to health practitioners through supporting professionalism and reducing burnout, and that ‘value‐based healthcare connects clinicians to their purpose as healers’ (p 682) [4].

## Conflicts of Interest

The author declares no conflicts of interest.

## Data Availability

Data sharing not applicable to this article as no datasets were generated or analysed during the current study.
